# Dietary prebiotics alter novel microbial dependent fecal metabolites that improve sleep

**DOI:** 10.1038/s41598-020-60679-y

**Published:** 2020-03-02

**Authors:** Robert S. Thompson, Fernando Vargas, Pieter C. Dorrestein, Maciej Chichlowski, Brian M. Berg, Monika Fleshner

**Affiliations:** 1 Department of Integrative Physiology, University of Colorado at Boulder, Boulder, CO 80309-0354 USA; 2 Center for Neuroscience, University of Colorado at Boulder, Boulder, CO 80309-0354 USA; 3 Division of Biological Sciences, University of California, San Diego, CA 92093 USA; 4 Collaborative Mass Spectrometry Innovation Center, Skaggs School of Pharmacy and Pharmaceutical Sciences, University of California, San Diego, CA 92093 USA; 5 Mead Johnson Pediatric Nutrition Institute, Evansville, IN 47712 USA

**Keywords:** Microbiome, Neurophysiology

## Abstract

Dietary prebiotics produce favorable changes in the commensal gut microbiome and reduce host vulnerability to stress-induced disruptions in complex behaviors such as sleep. The mechanisms for how prebiotics modulate stress physiology remain unclear; however, emerging evidence suggests that gut microbes and their metabolites may play a role. This study tested if stress and/or dietary prebiotics (Test diet) alter the fecal metabolome; and explored if these changes were related to sleep and/or gut microbial alpha diversity. Male F344 rats on either Test or Control diet were instrumented for electroencephalography biotelemetry measures of sleep/wake. After 5 weeks on diet, rats were either stressed or remained in home cages. Based on untargeted mass spectrometry and 16S rRNA gene sequencing, both stress and Test diet altered the fecal metabolome/microbiome. In addition, Test diet prevented the stress-induced reduction in microbial alpha diversity based on PD_Whole_Tree, which has been previously published. Network propagation analysis revealed that stress increased members of the neuroactive steroidal pregnane molecular family; and that Test diet reduced this effect. We also discovered links between sleep, alpha diversity, and pyrimidine, secondary bile acid, and neuroactive glucocorticoid/pregnanolone-type steroidal metabolites. These results reveal novel microbial-dependent metabolites that may modulate stress physiology and sleep.

## Introduction

Evidence in both the human and animal literatures suggest that stressor exposure can negatively impact sleep^1–4^ and the gut microbiota^5–7^. We have previously reported, for example, that male rats exposed to an acute stressor experience disruptions in sleep architecture, have flattened diurnal rhythmicity of core body temperature, learning deficits, express depressive-like behaviors, and altered gut microbial alpha diversity^4,8–10^. *Ad libitum* consumption of prebiotic diets, compared to nutrient/calorically matched control diets, prior to stressor exposure attenuates many of these effects^7,11^. In addition, ingestion of prebiotic diet also improves non-rapid eye movement (NREM) sleep, promotes increases in rapid eye movement (REM) sleep after stressor exposure (REM rebound), and prevents stress-induced reductions in gut microbial alpha diversity^7^.

The mechanisms for how prebiotic diet-induced changes in the gut microbiota impact stress physiology remain unclear; however, there is emerging evidence that gut microbial metabolites likely play a role^12,13^. Bacterial dependent metabolites, such as short chain fatty acids and secondary bile acids, can signal the brain through the blood and/or vagal afferents^14–16^. It is feasible, therefore, that gut microbial modulatory diets produce changes in the gut metabolome that signal the brain and impact complex brain functions. Fecal samples were collected both before and after stressor exposure and impacts on overall gut metabolomic structure were determined using untargeted mass spectrometry chromatography (LC-MS/MS). Those features driving the changes in metabolomic structure produced by diet and/or stress were then targeted for additional metabolomics standards initiative (MSI) identification and state-of-the-art network propagation analyses. Additional regression analyses explored if any of the identified gut metabolite changes were related to our previously reported effects on sleep and alpha diversity measured in fecal samples in the same rats [see^7^]. Discovering and identifying gut metabolites that are modulated by diet and/or stress and relate to sleep and microbial alpha diversity adds to our understanding of microbiota-gut-brain signaling and could hasten the development of health promoting microbiome therapeutics. Based on this previous work, we hypothesize that prebiotic diet will alter the fecal metabolome and that these metabolomics changes will be related to our previously reported stress-protective effects of prebiotic diet on sleep and microbiome alpha diversity.

## Results

### Test diet alters the fecal gut metabolome

**Figure 1:**
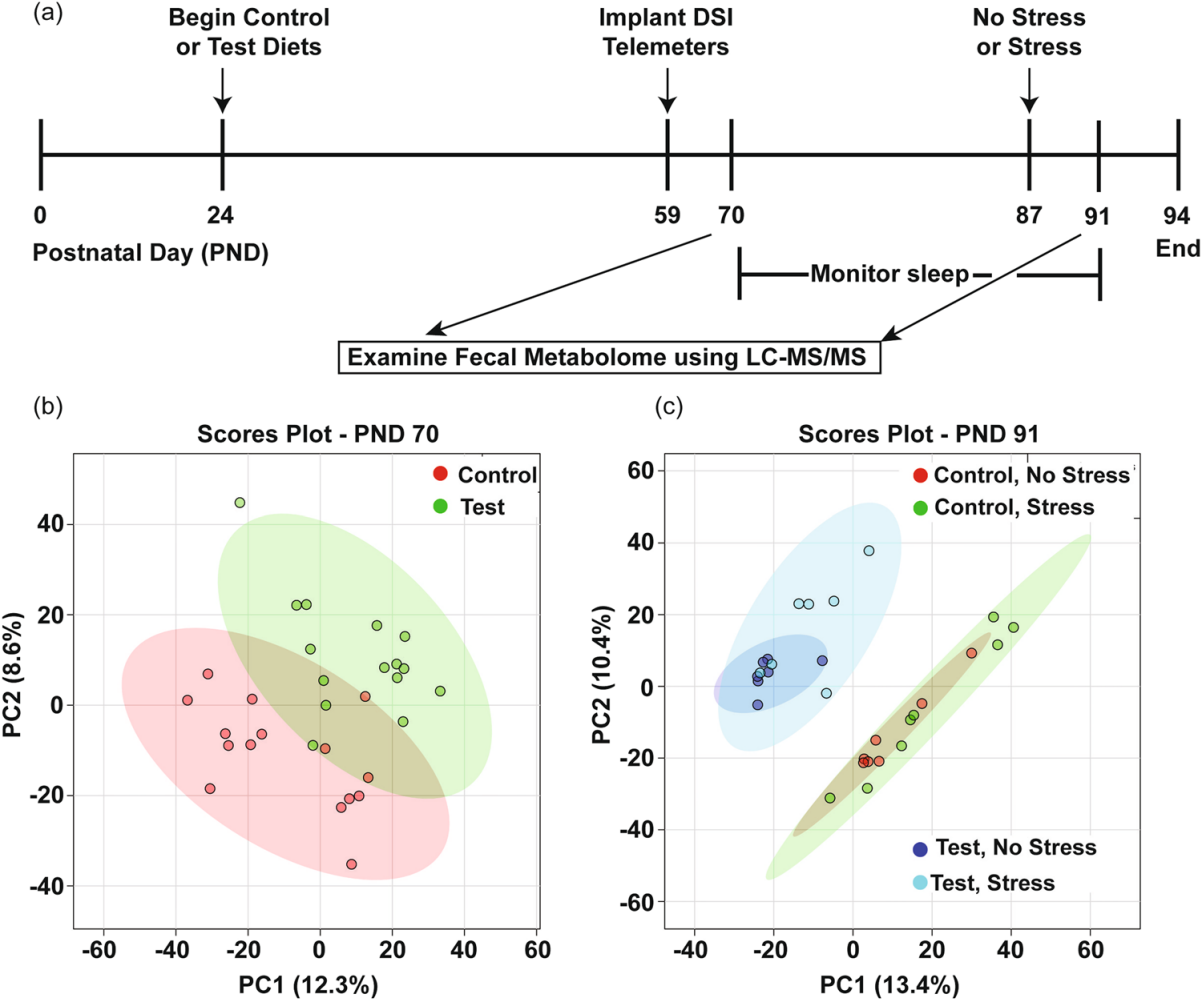
Adapted **(a)** timeline from Thompson *et al*. (2017), showing fecal samples were collected at PND 70 and at PND 91 for untargeted metabolomics analysis. **(b)** PC scores plot demonstrates separation by diet of the untargeted metabolome along component 1 and 2 at PND 70. **(c)** PC scores plot demonstrates large separation by diet of the untargeted metabolome along component 1 and 2 at PND 91. Shaded areas represent 95% confidence intervals. Abbreviations: Data Sciences International (DSI); Liquid chromatography-mass spectrometry (LC-MS/MS); Post-natal day (PND), Principal components (PC).

**Figure 2:**
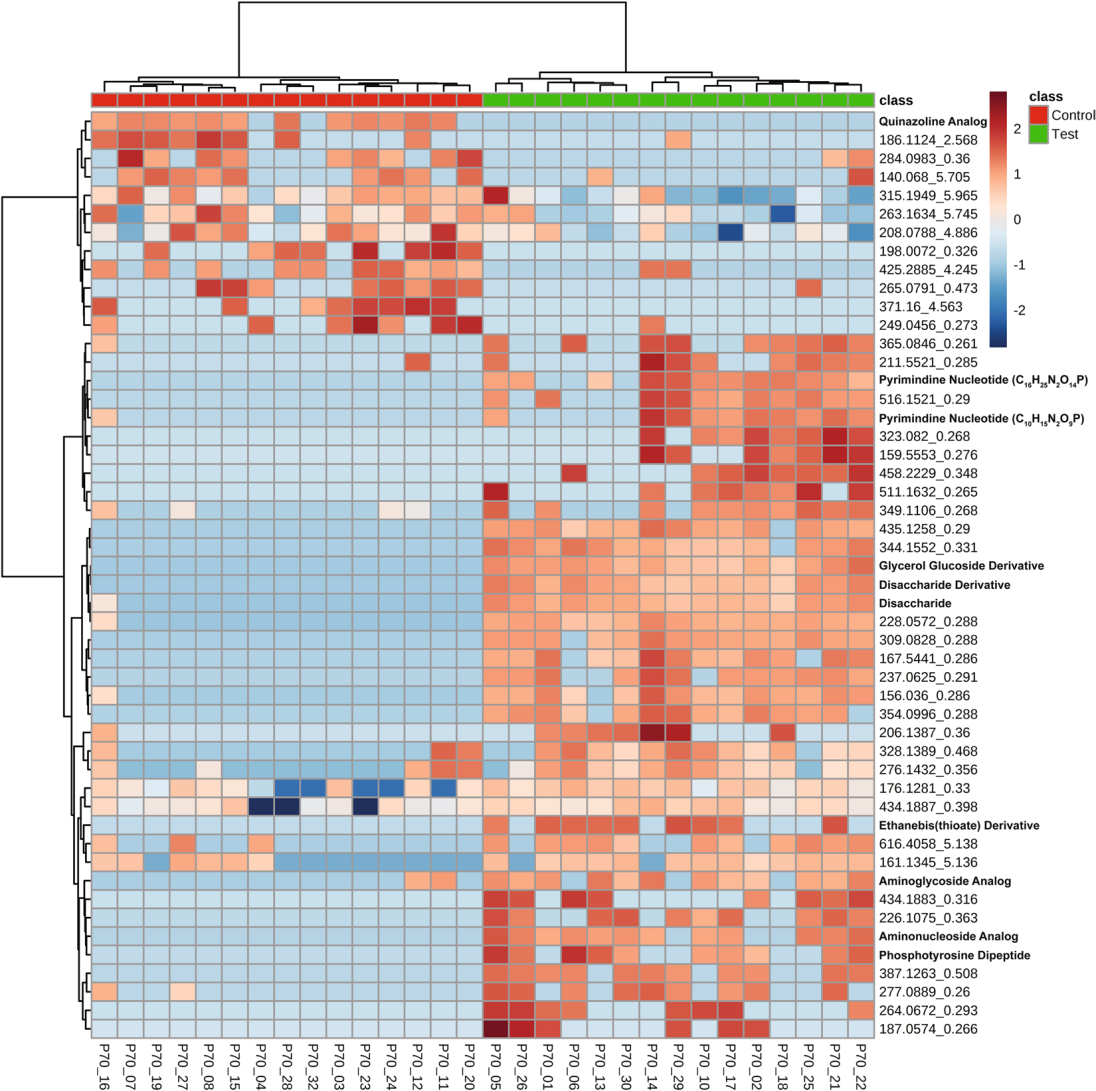
Heat map of the top 50 features that cluster (unsupervised) by control and Test diet measured in fecal samples collected on PND 70. Individual subjects are along the bottom, while features [mass/charge (m/z) retention times (min)] are listed to the right of the heat map. Approximately 12 features appear higher in the control compared to the Test diet while 38 appear higher in the Test diet.

At PND 70, the principal components (PC) plot demonstrates separation due to diet on the scores plot along component 1 and component 2 (Fig. 1a,b) based on the untargeted metabolomics analysis. These PC plots are a representation of all the metabolites measured (multidimensional) and compressed into two-dimensional space for simplification of interpretation. Shaded areas represent 95% confidence intervals (Fig. 1b,c). In a next step for PND 70, we then produced heat maps with the top 50 unidentified features and targeted (labeled) metabolites on the heat map showing unsupervised clustering by either control or Test diet at PND 70 (Fig. 2). For this heat map, individual subjects are listed along the bottom, while the features/metabolites [mass/charge (*m/z*)_retention times (*min*)] are listed to the right of the heat map.

**Figure 3:**
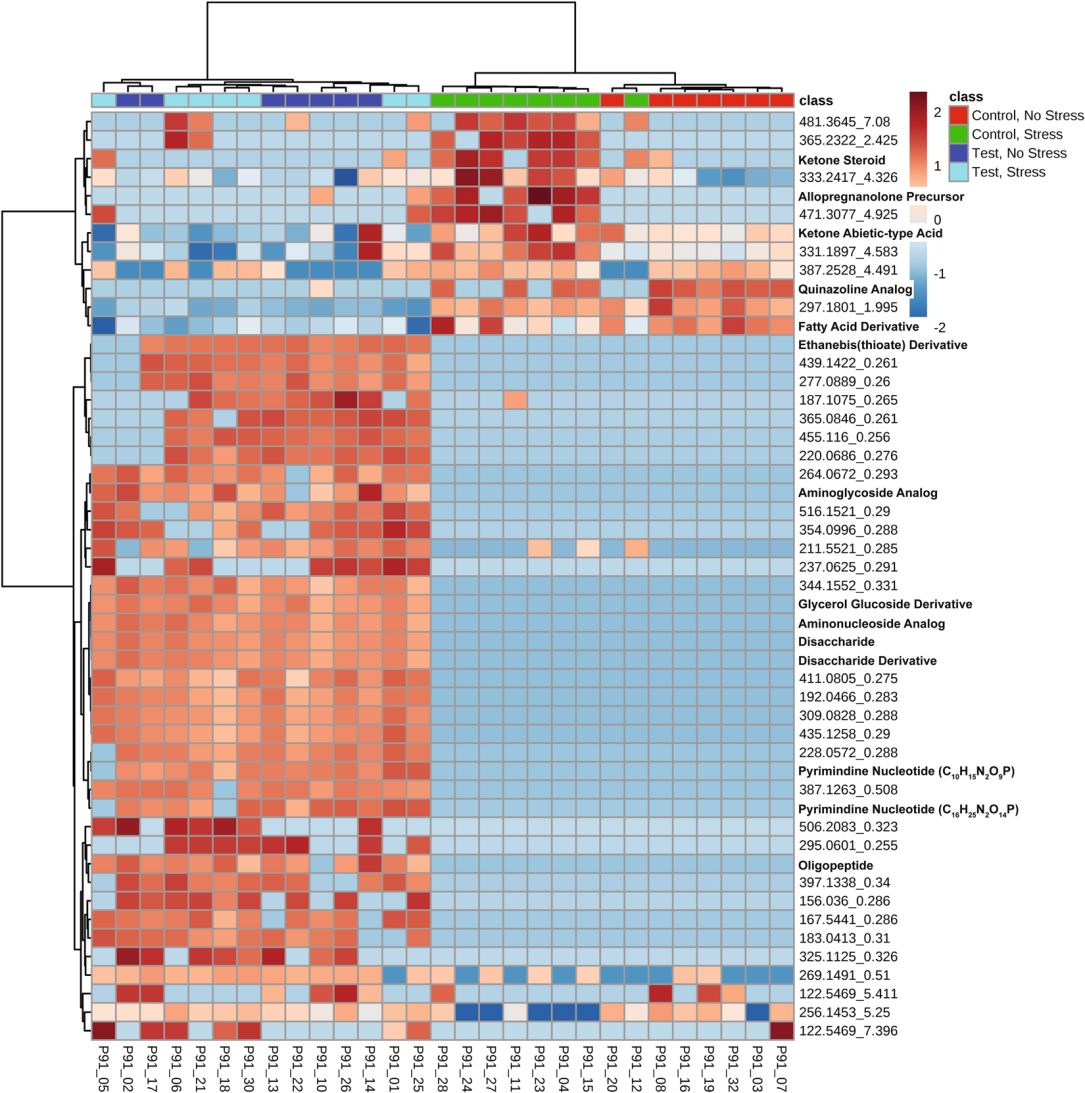
Heat map of the top 50 features that cluster (unsupervised) by diet and stressor exposure measured in fecal samples collected on PND 91. The features within the control diet clustered mostly by whether they were exposed to stress or not, suggesting a potential effect of stress exposure (upper right half of heat map). This effect of clustering by stress was absent in the Test diet group (upper left half of heat map). Overall, the features mostly clustered by diet, rather than stress, which is consistent with the PC plot. Individual subjects are along the bottom and features [mass/charge (*m/z*) retention times (*min*)] are listed to the right of the heat map.

Four days after stressor exposure at PND 91, diet impacted the fecal metabolome displayed on the PC plot mostly along component 1 (Fig. 1a,c). In a next step for this time point, we produced a heat map with the top 50 unidentified features and targeted metabolites on the heat map (Fig. 3) showing main clustering by diet and some clustering by stress within the control diet group.

PC plots and heat maps are used to broadly envision changes in large datasets. Detailed changes required additional analyses, using appropriate statistical methods as detailed below.

### Test diet and stress significantly alter specific ions and metabolites

**Figure 4:**
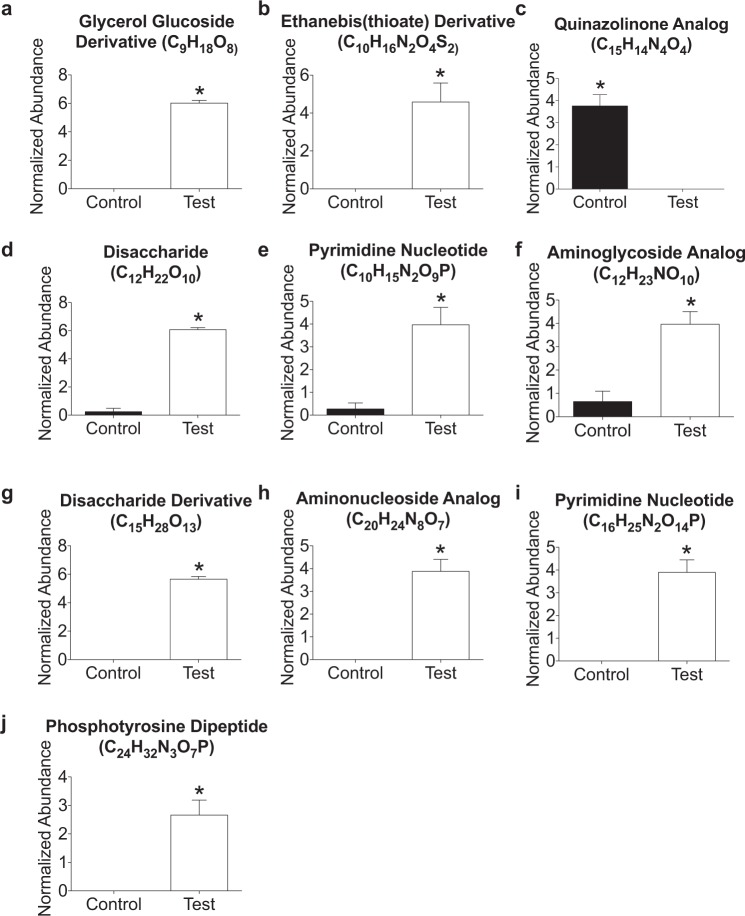
(**a**,**b**, **d**–**j)** Nine of the 10 identified metabolites were significantly higher in the Test diet when compared to the control diet at PND 70. **(c)** Alternatively, the quinazolinone analog was higher in the control diet when compared to the Test diet. (*p < 0.05 vs. control/test diet).

Volcano plot analysis (graphic shown in Supplemental Fig. 1) of the fecal metabolome on PND 70 revealed that 21 features were significantly impacted by diet (statistical results shown in Supplemental Table 2). All features except one were significantly higher in the Test diet when compared to the control diet. We identified 10 of these significant metabolites using CSIfinger ID achieving metabolomics standards initiative (MSI) level 3 annotation (Fig. 4a–j; Table 1). The following metabolites were significantly increased by Test diet: Glycerol Glucoside Derivative (Fig. 4a), Ethanebis(thioate) Derivative (Fig. 4b), Disaccharide (Fig. 4d), Pyrimidine Nucleotide 1 (Fig. 4e), Aminoglycoside Analog (Fig. 4f), Disaccharide Derivative (Fig. 4g), Aminonucleoside Analog (Fig. 4h), Pyrimidine Nucleotide 2 (Fig. 4i), Phosphotyrosine Dipeptide (Fig. 4j). Only the Quinazolinone Analog was significantly higher in the control diet (Fig. 4c).

**Table 1:** Metabolites that were identified using SIRIUS and CSIfinger ID.

CSIfinger ID predicted family	Figure	row ID	row m/z	retention time	MF(SIRIUS)	Tree Score	ISOTOPE	Peaks	MSI
Fatty Acid Derivative	5 m	69	190.1436725	0.354737288	C_9_H_19_NO_3_	109.0061428	3.215039989	35	3
Glycerol Glucoside Derivative	4a, 5a	110	255.1077526	0.329195152	C_9_H_18_O_8_	176.2721696	5.50958751	46	3
Ethanebis(thioate) Derivative	4b, 5b, 6c	20	293.0628507	0.255751818	C_10_H_16_N_2_O_4_S_2_	151.43	0	34	3
Quinazolinone Analog	4c, 5 l	1678	315.1109357	0.354012147	C_15_H_14_N_4_O_4_	149.8741117	0	44	3
Ketone Abietic-type Acid	5n	38	315.1948988	5.966663559	C_20_H_26_O_3_	47.63533148	0	25	3
Ketone Steroid	5k, 6c	175	317.2469707	5.065049718	C_21_H_32_O_2_	125.0395243	3.410851867	51	3
Disaccharide	4d, 5c	114	327.1282495	0.333138636	C_12_H_22_O_10_	182.4499773	3.476393104	54	3
Pyrimidine Nucleotide	4e, 5d	58	339.060153	0.280809322	C_10_H_15_N_2_O_9_P	77.00574093	2.785892451	40	3
Aminoglycoside Analog	4 f, 5e	244	342.1397425	0.305590805	C_12_H_23_NO_10_	151.636548	0	55	3
Allopregnanolone Precursor	5j, 6d	379	349.2369875	4.085015819	C_21_H_32_O_4_	189.93	3.8	55	3
Disaccharide Derivative	4 g, 5 f	160	417.1602149	0.319994737	C_15_H_28_O_13_	191.2993464	3.629846586	52	3
Oligopeptide	5 g	107	434.1887824	0.399667232	C_16_H_27_N_5_O_9_	53.33577828	3.458464457	36	3
Aminonucleoside Analog	4 h, 5 h	222	489.1809597	0.334691441	C_20_H_24_N_8_O_7_	171.5941177	2.036780536	53	3
Pyrimidine Nucleotide	4i, 5i, 6b	128	501.1125744	0.27855787	C_16_H_25_N_2_O_14_P	74.72	0	48	3
Phosphotyrosine Dipeptide	4j	328	506.2087682	0.332613333	C_24_H_32_N_3_O_7_P	151.2632419	0	51	3

See additional classification specifications for these features in Supplemental Table 1.

**Figure 5:**
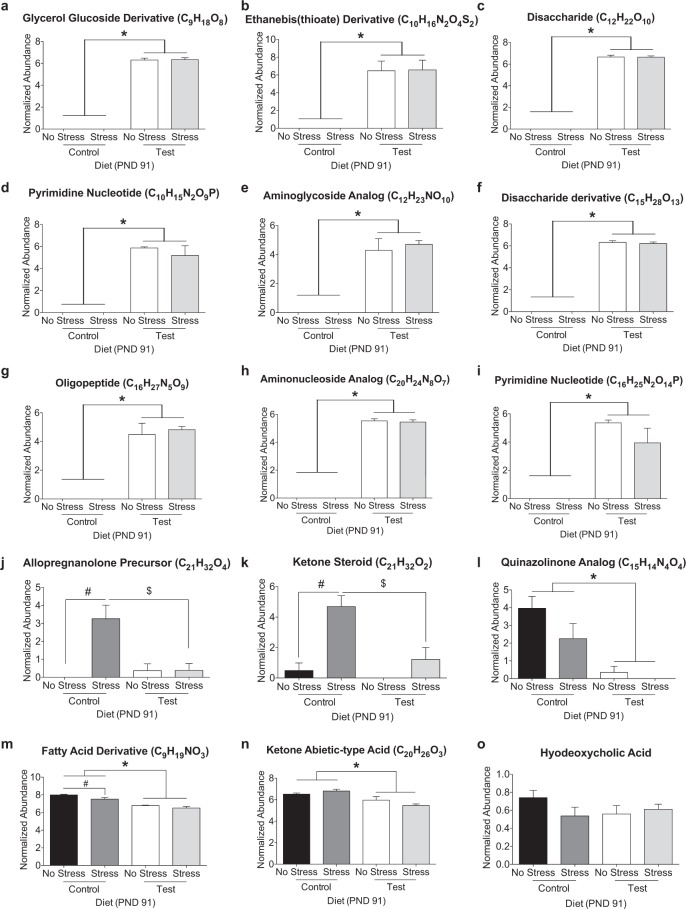
The metabolites in (**a**–**i)** were significantly higher in Test diet compared to control diet. The stress-induced increase in the **(j)** allopregnanolone precursor and **(k)** ketone steroid was attenuated by Test diet. **(l)** Quinazolinone analog was higher in the control diet. Both **(m)** the Fatty Acid Derivative and **(n)** the Ketone Abietic-type Acid were lower in the test diet, whereas stress decreased the Fatty Acid Derivative in the control diet group only. Finally, there was no significant effect of either diet or stress on **(o)** the secondary bile acid hyodeoxycholic acid. (*p < 0.05 vs. control diet – main effect; ^#^p < 0.05 as compared to no stress; ^$^p < 0.05 control stress vs. Test stress).

Analyses of the fecal metabolome on PND 91 revealed 36 unidentified features as significantly different between groups (two-way ANOVA p < 0.05, FDR p < 0.05; Supplemental Fig. 2; Supplemental Table 3). There were 28 of 36 features that were higher in the Test diet when compared to control diet, with no effect of stress. The remaining 8 of 36 features were altered by both diet and/or stress. We identified 14 of these features using CSIfinger ID achieving metabolomics standards initiative (MSI) level 3 annotation (Fig. 5a–n; Table 1). The following features were significantly increased by Test diet with no effect of stress exposure: Glycerol Glucoside Derivative (Fig. 5a), Ethanebis(thioate) Derivative (Fig. 5b), Disaccharide (Fig. 5c), Pyrimidine Nucleotide 1 (Fig. 5d), Aminoglycoside Analog (Fig. 5e), Disaccharide Derivative (Fig. 5f), Oligopeptide (Fig. 5g), Aminonucleoside Analog (Fig. 5h), Pyrimidine Nucleotide 2 (Fig. 5i). There was a significant effect of stress on the Allopregnanolone Precursor (Fig. 5j) and the Ketone Steroid (Fig. 5k) that was attenuated in the Test diet stress group. The Quinazolinone Analog (Fig. 5l), the Fatty Acid Derivative (Fig. 5m), and the Ketone Abietic-type Acid (Fig. 5n) were significantly lower in the Test diet. The Fatty Acid Derivative (Fig. 5m) was significantly reduced in the control diet stress group. See Fig. 5 for specific effects and post hoc analyses on individual metabolites.

The following metabolites were identified at both time points: Glycerol Glucoside Derivative (C_9_H_18_O_8_); Ethanbis(thioate) Derivative (C_10_H_16_N_2_O_4_S_2_); Disaccharide (C_12_H_22_O_10_); Pyrimidine Nucleotide (C_10_H_15_N_2_O_9_P); Aminoglycoside Analog (C_12_H_23_NO_10_); Disaccharide Derivative (C_15_H_28_O_13_); Aminonucleoside Analog (C_20_H_24_N_8_O_7_); Pyrimidine Nucleotide (C_16_H_25_N_2_O_14_P). All of these metabolites were elevated by Test diet at both time points PND 70 and PND 91. Alternatively, the Quinazolinone Analog (C_15_H_14_N_4_O_4_) was significantly elevated in the control diet at both time points while stress reduced this metabolite in the control diet at time point PND 91 (see Fig. 5l for results of post-hoc analysis).

### Network analysis of pregnane neuroactive steroids

Based on the GNPS database, the stress-responsive allopregnanolone precursor in Fig. 5j is a spectral match to the neuroactive steroid 5.alpha.-Pregnane-3.alpha., 21-diol-11, 20-dione. The calculated mass is within 5 ppm and the MS/MS spectrum is a nearly identical match (Supplemental Fig. 3). The spectral match to 5.alpha.-Pregnane-3.alpha., 21-diol-11, 20-dione was at level 3, according to MSI^17^ and confirms that this allopregnanolone precursor matches the pregnane neuroactive steroidal molecular family. In order to further elucidate this pregnane metabolite class in our fecal samples, we ran state-of-the-art network propagation analysis^18^ to classify the allopregnanolone precursor (5.alpha.-Pregnane-3.alpha., 21-diol-11, 20-dione) with familial molecules of similar structure.

**Figure 6:**
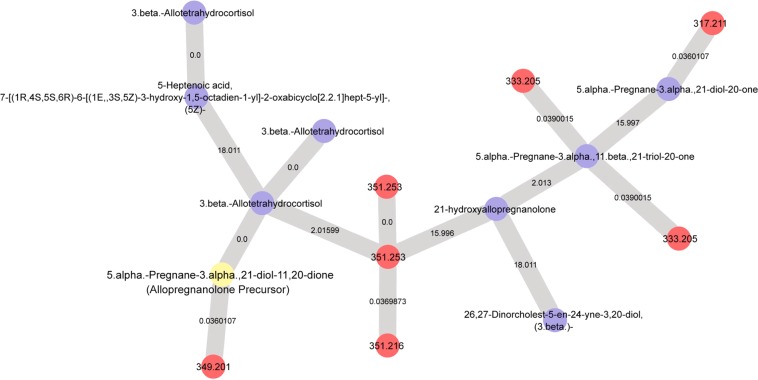
Molecular network analysis reveals several metabolites that are structurally related to the Allopregnanolone Precursor (yellow node). Nodes are labeled according to their precursor mass or *m/z* (red nodes) or their GNPS spectral library match (blue nodes). Nodes are connected via edges where they are labeled based on the *m/z* difference between nodes.

The resulting network propagation analysis is depicted in Fig. 6. In this network, the nodes are labeled according to their precursor mass or *m/z* (red nodes) or their GNPS spectral library match (blue nodes). Nodes are connected via edges (gray lines) where they are labeled based on the *m/z* difference between nodes. The allopregnanolone precursor (yellow in Fig. 6) was structurally related to several other metabolites demonstrated by the connected edges within the network. Some specific examples of metabolites that were matched based on the GNPS spectral library (blue nodes) include: 3.beta.-Allotetrahydrocortisol; 21-hydroxyallopregnanolone; 5.alpha.-Pregnane-3.alpha.,11.beta., 21-triol-20-one; and 5.alpha.-Pregnane-3.alpha., 21-diol-20-one.

The allopregnanolone precursor (yellow in Fig. 6) belongs to a neuroactive steroidal molecular family and molecular network for glucocorticoid and pregnanolone-type steroids (MSI Level 3 spectral match), adding confidence that the Allopregnanolone precursor depicted in Fig. 5j is correctly annotated as 5.alpha.-Pregnane-3.alpha., 21-diol-11, 20-dione.

### Relationships between identified metabolites, sleep architecture, and microbiome alpha diversity

**Figure 7:**
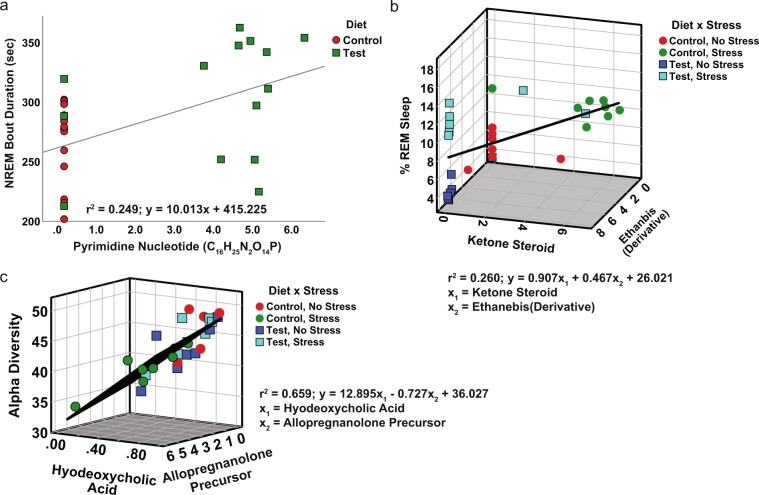
(**a**) Regression analysis demonstrating a significant, linear relationship between the Pyrimidine Nucleotide and NREM sleep bout duration at PND 70. (**b)** Regression demonstrating a significant relationship between the stress responsive Ketone Steroid + the Ethanebis(thioate) Derivative and REM sleep rebound after stress exposure. (**c)** Regression demonstrating a significant relationship between alpha diversity (PD_Whole_Tree) and Hyodeoxycholic acid + the Allopregnanolone Precursor.

We previously reported that rats fed Test diet had an increase in NREM sleep bout durations, when compared to those fed control diet [Fig. 4B,D from^7^]. Potential relationships between the observed NREM sleep changes at PND 71,72 from our previous findings and the identified fecal metabolome at PND 70 (Fig. 4) were explored using stepwise multiple regression analysis. We performed a regression analysis of all of the identified metabolites as well as bacterial phylum with NREM sleep to see if there were any relationships between NREM sleep and any of these variables at PND 70. This analysis revealed a significant linear relationship between Pyrimidine Nucleotide (C_16_H_25_N_2_O_14_P) depicted in Fig. 4i and NREM sleep (F_(1, 28)_ = 8.939; p = 0.006; adj. r^2^ = 0.249; y = 10.013x + 415.225; Fig. 7a).

We also previously published that rats eating Test diet had increased REM sleep rebound following acute stress exposure [Fig. 5 from^7^]. It may be possible that a relationship exists between the increased REM sleep rebound after stress exposure and the gut metabolome. Similarly, we performed a regression analysis of all of the identified metabolites as well as bacterial phylum with REM sleep to see if there were any relationships between REM sleep and any of these variables at PND 91. Indeed, stepwise multiple regression revealed that two identified features at PND 91 were related to REM sleep after stress at PND 87 (F_(2, 27)_ = 5.736; p = 0.009; adj. r^2^ = 0.260; y = 0.907x_1_ + 0.467x_2_ + 26.021; Fig. 7b). The first feature x_1_ is the stress responsive Ketone Steroid (Fig. 5k) and the second x_2_ is Ethanebis(thioate) Derivative (Fig. 5b).

Test diet attenuated the stress induced increase in alpha diversity of the gut microbiome [Fig. 7C (PD_Whole_Tree) from^7^] and thus we examined potential relationships between the identified gut metabolites and alpha diversity. We performed a separate regression analysis between all of the identified metabolites as well as bacterial phylum with alpha diversity to examine any relationships between these variables at PND 91. There was a significant relationship between two identified metabolites at PND 91 and alpha diversity (F_(2, 27)_ = 27.092; p < 0.001; adj. r^2^ = 0.659; y = 12.895x_1_ − 0.727x_2_ + 36.027; Fig. 7c). The two metabolites are x_1_ = Hyodeoxycholic acid and x_2_ = Alloprenanolone Precursor (yellow in Fig. 6; Fig. 5j).

## Discussion

Ingestion of a prebiotic diet (Test diet) improves undisturbed non-rapid eye movement (NREM) sleep, promotes REM sleep rebound after stress exposure, and prevents stress-induced reductions in gut microbial alpha diversity^7^. The results from the current report demonstrate that Test diet also modulates the fecal metabolome community, increases specific metabolites (fatty acids, sugars, steroids, nucleotides) several of which are consistently elevated across time, and prebiotic-induced sleep improvements are related to several fecal metabolites. This report along with prior studies^7,9,19,20^ suggests that fecal metabolites are an important effector arm of the microbiota-gut-brain axis and adds to emerging evidence linking metabolomics and sleep physiology^21^.

Stressor exposure affected the fecal metabolome differently in the control vs. the Test diet. Test diet attenuated the stress-induced increase in the Allopregnanolone Precursor, the Ketone Steroid, as well as two other unidentified metabolite features. These small fecal metabolites belong to a family of endogenous metabolites of corticosterone/progesterone. Network propagation analysis^18^ revealed several other potential metabolites in the family. One such metabolite is 5.alpha.-Pregnane-3.alpha., 11.beta., 21-triol-20-one, which has been reported to block voltage-dependent calcium channels^22^ and another is 5.alpha.-Pregnane-3.alpha., 21-diol-20-one, better known as allotetrahydrodeoxycorticosterone, which is also a neuroactive steroid that potentiates GABAergic inhibition^23^. This metabolite has also recently been linked with reduced sleep quality during pregnancy^24^ and is involved in the acute stress response in rodents^25–27^. These data suggest that stress may affect neuroactive steroid signaling in the gut, which is attenuated by a prebiotic diet. It could be that the negative consequences of stress exposure, in part, are mitigated through gut microbial modulatory substrates such as dietary prebiotics. This idea is congruent with a role for dysregulated neuroactive steroid signaling in stress-related psychiatric disorders^28^. It is important to note that although fecal microbiome/metabolome likely reflects intestinal composition, there are examples in the literature that challenge this idea^29,30^.

We discovered several novel fecal metabolites that were related to measures of sleep suggesting a potential link between the fecal metabolome and sleep physiology. Specifically, improved NREM sleep was related to a Pyrimidine Nucleotide at PND 70. This relationship is consistent with prior literature examining a role for pyrimidine metabolism in sleep^31^. CSIfinger ID gives the molecular formula for this Pyrimidine Nucleotide as C_16_H_25_N_2_O_14_P, but no known reference standard is yet available for this byproduct of pyrimidine metabolism, however based on the proposed molecular formula it may be involved in uracil metabolism, a metabolite known to increase NREM sleep^32,33^. Similarly, the previously reported enhanced REM sleep rebound following stress^7^ was significantly related to the stress-responsive Ketone Steroid (C_21_H_32_O_2_) + Ethanebis(thioate) Derivative (C_10_H_16_N_2_O_4_S_2_). Based on the molecular formula for the Ketone Steroid this molecule is a steroid derivative with a pregnane skeleton, however we verified using a standard that this molecule is not pregnenolone, per se, rather likely related to pregnenolone. Given that this class of molecules has been implicated in the regulation of sleep physiology^34^; it is possible that the Ketone Steroid detected in this study is a novel sleep modulatory fecal metabolite. The second factor in the relationship with REM sleep was an Ethanebis(thioate) Derivative. Based on the molecular formula this gut metabolite is still unannotated and represents a novel potential molecule potentially involved in sleep. Although the mechanisms for how changes in fecal metabolites impact complex brain functions such as sleep remain unknown, these data reveal novel relationships between fecal pyrimidine metabolism, stress responsive fecal neuroactive steroids, and sleep physiology. Previous studies have linked both plasma/urine metabolites with sleep physiology^35–38^, but to the best of our knowledge this is the first study to relate changes in fecal metabolome produced after ingestion of gut microbial modulatory substrates with sleep physiology.

The gut metabolome and gut microbiome are linked through gut microbial metabolism. Our findings help clarify this relationship, such that microbiome alpha diversity was related to a fecal hyodeoxycholic acid and the Allopregnanolone Precursor. Hyodeoxycholic acid is a secondary bile acid that is dependent on the gut microbiota and impacts health and disease^39–41^. It is possible, therefore, that some of the health benefits often associated with elevated gut microbial alpha diversity^7,42,43^ are due to modulation of secondary bile acids. This idea is further supported by our data demonstrating that a Fatty Acid Derivative is modulated by both stress and Test diet. The second metabolite in the regression is the Allopregnanolone Precursor, which belongs to a molecular class of neuroactive steroids acting at GABA receptors^25^. The relationship reported here between alpha diversity, secondary bile acids, and a neuroactive gut metabolite strengthens the idea that the fecal metabolome is linked through gut microbial metabolism and taken together with other results suggests that these play a role in modulating stress and sleep physiology. This work represents an important step towards uncovering the potential mechanisms underlying health promoting gut microbial modulating substrates. There remain, however, many currently unidentifiable features that were modulated by prebiotic and/or stress. With continued improvements in available reference samples^44^, future work will expand the identification of potential gut derived biomolecular pathways and could facilitate the discovery of additional novel molecules capable of impacting physiology and complex behavior.

## Materials and Methods

### Animals

Adult male F344 rats (n = 52, Harlan Laboratories) were housed with controlled temperature and humidity and all procedures were approved by the University of Colorado Animal Care and Use as previously described in detail^7^. Briefly, animals weighed 40–50 g upon arrival at post-natal day (PND) 24 and were maintained on a 12:12 h light/dark cycle. All rats were housed in Nalgene Plexiglas cages and were placed on control or Test diet *(ad libitum)* upon arrival at PND 24. Rats were allowed to acclimate to housing conditions for 1 week prior to study initiation. As previously described^7^, upon arrival rats were double housed due to the young age. After biotelemetry implantation, rats were single housed in order to acquire accurate telemetry signals from each rat. All fecal samples were collected from individually housed rodents for fecal metabolomics analysis. No differences in body weight for food consumption were found and there were 14–15 animals per group (diet) at PND day 70 data and 7–8 animals per group (diet x stress) for the PND 91 data (Fig. 1a).

### Experimental design

The experimental design is depicted in Fig. 1 and adapted from Thompson *et al*., 2017. Fecal samples used for gut metabolomics analysis were collected at the same time points as those used for gut microbiome analyses previously reported^7^. Animals consumed diet for 7 weeks prior to the first fecal sample collection at PND 70 and then for 11 weeks. Animals were then exposed to inescapable tail shock stress or not, and four days following inescapable tail shock stress another fecal sample was taken at PND 91 (Fig. 1a).

### Control and test diet

Test diet contained the following gut microbial modulatory nutrients, which are absent from control diet: galactooligosaccharides (GOS 21.23 g (total)/Kg [7.00 g (active)]/Kg]; FrieslandCampina, Zwolle, Netherlands), polydextrose (PDX, 6.58 g (active)/Kg; Danisco, Terre Haute, IN, USA), lactoferrin (LAC, 1.86 g/Kg; Tatua Cooperative Dairy Company, Morrinsville, New Zealand), and whey protein concentrate milk fat globular membrane protein-10 (MFGM, 15.9 g/Kg; Arla Food Ingredients, Aarhus, Denmark). GOS is a non-absorbable complex carbohydrate derived from the enzymatic breakdown of lactose; and PDX is a processed polymer derived from glucose and classified as a soluble fiber by the US Food and Drug Administration. Both GOS and PDX are classified as prebiotic substrates because they are (1) not hydrolyzed or absorbed in the stomach or small intestine; (2) selective substrates for beneficial commensal bacteria in the colon, such as *Lactobacillus* spp.; and (3) induce beneficial luminal/systemic effects within the host^45–49^. LAC impacts the gut microbiota through microbiostatic and antimicrobial activity^50,51^, and MFGM alters antimicrobial activity^52^ as well as the microbiota^53,54^. Animals were fed either control or Test diet and experimenters were blind to diet type. The diets were formulated by Mead Johnson Nutrition (MJN, Evansville IN, USA) based on AIN-93G specifications and were isocaloric with similar carbohydrate, protein, fat, vitamin, and mineral levels.

### Fecal sample collection

Stool samples were collected as previously described^7^. Briefly, rats were placed into a sterile cage until defecation (<10-min) where samples were collected on ice and rats were immediately returned to the home cage. Samples were then transferred and stored in a −80 C freezer for untargeted metabolomics analysis.

### Stress protocol

Unpredictable and inescapable tail shock is a well-characterized laboratory stressor that robustly and reproducibly produces depression/anxiety-like behavior, elevates corticosterone, and disrupts diurnal physiology, sleep and the microbiome^3,4,7,8,55,56^. In brief, rats were placed in Plexiglas restraining tubes (23.4 cm long and 7.0 cm in diameter) and exposed to 100, 5-s, 1.5 mA inescapable tail shocks (Stress). Shocks were delivered at random with an average interval of 60-s between shocks and occurred during the inactive (light) cycle between ~0800 and 1100 h. After exposure to inescapable tail shock rats were immediately returned to their home cages. Animals that were not exposed to the stressor (No Stress), remained undisturbed in their home cages.

### Sleep measures

Sleep was measured using *in vivo* biotelemetry, as previously described^4,8,57^. The complete sleep results from these rats have been previously reported in^7^. In brief, the F40-EET biotelemetry transmitters (Data Sciences International, St. Paul Minnesota) were implanted into animals and the electroencephalographic (EEG) insulated leads were passed subcutaneously to the base of the skull, where they were attached to stainless steel screws (Plastics One Inc.) and served as EEG recording electrodes. Biotelemetry recordings of EEG were acquired using Dataquest ART 4.3 Gold Acquisition/Analysis Software (Data Sciences International, St. Paul, MN) and sleep/wake cycles were scored using the automated Neuroscore 2.1.0 software (Data Sciences International, St. Paul, MN). After sleep recordings were autoscored, they were corrected for accuracy by an observer blind to the experimental treatment of each animal.

The current study used two measures of sleep, NREM bout duration and % REM. NREM and REM measures were derived from the trace EEG signal after fast Fourier Transformation (FFT), yielding spectra between 0.5 and 30 Hz in 0.5-Hz frequency bins. NREM sleep was identified by increased absolute EEG amplitude with integrated values for the delta frequency band (0.5–4.5 Hz) being greater than those for the theta frequency band (6.0–9.0 Hz). REM sleep was characterized by low amplitude EEG with integrated values for the delta frequency band less than those for the theta frequency band. Time spent in REM was calculated as a percentage (%) of time spent in a specific behavioral state per hour. Average bout durations per hour of NREM were also calculated. Bout durations were defined by any change in sleep/wake state for 10 seconds (i.e., an NREM bout was defined based on the appearance of 10-sec epoch or longer of NREM and the end of that epoch was defined as the appearance of any 10-sec epoch of either REM or WAKE).

### 16S rRNA gene sequencing and microbial alpha diversity analyses

Fecal samples were previously collected at PND 70 and PND 91 and sequenced as described^7^. These same fecal samples were used for metabolomics analysis. In brief, after purification and precipitation to remove polymerase chain reaction (PCR) artifacts, samples were exposed to multiple sequencing on an Illumina Genome Analyzer IIx. Operational taxonomic units (OTUs) were picked using a ‘closed reference’ approach^58^. GreenGenes May 2013 version was the reference database used^59^, and all sequence processing was done with QIIME v 1.8.0^60^ using the UCLUST algorithm^61^. Taxonomy and phylogeny were taken from the GreenGenes reference collection. The current experiment generated 14,207,155 sequences, where 11,016,354 were discarded because of uncorrectable barcode errors, of which 6,481 were too short to read (based on default parameters set in QIIME script ‘split_libraries_fastq.py’) and the remaining 3,190,801 sequences were used. The resulting OTU table was rarefied at 7400 sequences/sample to correct for uneven sequencing depth due to amplification differences between samples. Alpha diversity for this manuscript was measured using species richness (PD_Whole_Tree). This measure captures phylogenetic diversity for a given sample^62^.

### Metabolomics

#### Sample information – LC – MS/MS

A subset of frozen fecal samples was transferred via dry ice to the University of California, San Diego for metabolomic analysis. Fecal samples were stored in 1.5 mL centrifuge tubes at −80 °C prior to extractions. Sample ID’s were manually uploaded into an electronic spreadsheet and subsequently used to assign filenames during LC-MS/MS data acquisition. All solvents used for the metabolomic analysis were of LC-MS grade.

#### Fecal pellet extraction – LC – MS/MS

This method was adapted from a previously published protocol^63^. Fecal pellets were weighed to 50.0 +/− 2 mg wet weight and transferred to 2.0 mL round bottom microcentrifuge tubes (Qiagen Catalog# 990381) for metabolite extractions. A clean stainless-steel bead (Qiagen Catalog# 69989) and 1.5 mL chilled extraction solvent (50% MeOH) was added to each sample. The samples were then homogenized for 5 min at 25 Hz using a TissueLyser II system (Qiagen Catalog# 85300) and allowed to incubate for 20 min at −20 °C. The fecal homogenates were then centrifuged at 14000 rpm for 15 min at 4 °C. 1.2 mL aliquots were then transferred into Nunc 2.0 mL DeepWell plate (Thermo Catalog# 278743) and frozen at −80 °C prior to lyophilization using a FreeZone 4.5 L Benchtop Freeze Dryer with Centrivap Concentrator (Labconco). Wells were resuspended with 200 µL of resuspension solvent (50% MeOH spiked with 2.0 µM sulfadimethoxine), vortexed for 30 secs, and centrifuged at 2000 rpm for 15 min at 4 °C. 150 µL of the supernatant was transferred into a 96-well plate and maintained at 4 °C prior to LC-MS analysis. A resuspension solvent QC and a six standard mix QC (50% MeOH spiked with 1.0 µM Sulfamethazine, 1.0 µM Sulfamethizole, 1.0 µM Sulfachloropyridazine, 1.0 µM Amitrypline, and 1.0 µM Coumarin 314) was run every12^th^ sample to assess sample background, carry over, chromatography behavior, peak picking and plate effects.

#### LC-MS/MS parameters

Fecal extracts were analyzed using an ultra-high performance liquid chromatography system (Vanquish, Thermo) coupled to a hybrid quadrupole-Orbitrap mass spectrometer (Q-Exactive, Thermo) fitted with a HESI probe. Reverse phase chromatographic separation was achieved using a Kinetex C18 1.7 µm, 100 Å, 50 × 2.1 mm column (Phenomenex) held at 40 °C with a flow rate of 0.5 mL/min. 5.0 µL aliquots were injected per sample/QC. The mobile phase used was (A) 0.1% formic acid in water and (B) 0.1% formic acid in acetonitrile. The elution gradient was: 5% B for 1 min, increased to 100% B in the next 8 min, held at 100% B for two min, returned to 5.0% B in 0.5 min, equilibrated at 5.0% B for two min. Positive electrospray ionization parameters were: sheath gas flow rate of 52 (arb. units), aux gas flow rate of 14 (arb. units), sweep gas flow rate of 3 (arb. units), spray voltage of 3.5 kV, capillary temperature of 270 °C, S-Lens RF level of 50 (arb. units), and aux gas heater temperature of 435 °C. Negative electrospray ionization parameters were: sheath gas flow rate of 52 (arb. units), aux gas flow rate of 14 (arb. units), sweep gas flow rate of 3 (arb. units), spray voltage of 2.5 kV, capillary temperature of 270 °C, S-Lens RF level of 50 (arb. units), and aux gas heater temperature of 435 °C. MS data was acquired using a data dependent acquisition method with a resolution of 35,000 in MS^1^ and 17,000 in MS^2^. An MS^1^ scan from 100–1500 *m/z* was followed by an MS^2^ scan, produced by collision induced disassociation, of the five most abundant ions from the prior MS^1^ scan.

#### Data processing and analysis

The orbitrap files (.raw) were exported to mzXML files using MSConvert^64^. Feature detection of the MS^1^ data was performed using MZmine2^65^, parameters can be found in Supplemental Table 1, which generated a data matrix of MS^1^ features (*m/z* and retention time) and peak area. Each feature in a given sample was normalized against the spiked internal standard, to remove any spray variation across runs, followed by normalization of each sample by the row sum of its features. The twice-normalized data matrix was used for all subsequent statistical analysis. The SIRIUS export module found within MZmine was used to create.mgf files for molecular formula annotation and molecular structure prediction in the SIRIUS desktop software.

#### Global natural products social molecular networking (gnps) job parameters

Molecular networking was ran using networking parameters that yielded a false-discovery rate (FDR) of annotation, using Passatuto, for spectral matches against reference libraries of 1% (Supplemental Fig. 4). A molecular network was created using the online workflow at GNPS^66^. The data was then clustered with MS-Cluster with a parent mass tolerance of 0.05 Da and a MS/MS fragment ion tolerance of 0.05 Da to create consensus spectra. Further, consensus spectra that contained less than 2 spectra were discarded. A network was then created where edges were filtered to have a cosine score above 0.6 and more than 6 matched peaks. Further edges between two nodes were kept in the network if and only if each of the nodes appeared in each other’s respective top 10 most similar nodes. The spectra in the network were then searched against GNPS’ spectral libraries. All matches kept between network spectra and library spectra were required to have a score above 0.52 and at least 6 matched peaks^67^. The molecular networking job can be accessed using the following GNPS positive mode link: https://gnps.ucsd.edu/ProteoSAFe/status.jsp?task=92166ef840924b078fed960323cbd558.

#### Standards run for bile acids

Primary, secondary, conjugated and unconjugated bile acids were purchased and used for level 1 identification of some of our unknown molecules. Standards were solubilized to a final concentration of 10uM in 50% MeOH prior to LC-MS/MS injection.

#### Metabolite annotation

Metabolites were annotated following the guidelines established by the 2007 metabolomics Standards initiative (MSI). Accurate mass with retention time alignment and MS/MS fragmentation pattern between a metabolite of interest and a chemical reference standard was used for all MSI level 1 annotations. Accurate mass and MS/MS fragmentation pattern between a metabolite of interest and a reference library, via GNPS, was used for all MSI level 2 annotations. SIRIUS 4.0.1 was used for molecular formula annotation (MSI level 4) and CSI:FingerID, via SIRIUS 4.0.1, was used for molecular structure predication (MSI level 3)^68,69^.

### Statistical analysis

Metabolomics data were visualized and analyzed using metaboanalyst open source www.metaboanalyst.ca built on R statistical software^70–74^ and all other analyses were performed in SPSS version 25. All features were log transformed prior to further analysis. Principal components analysis was used to reduce the high dimensionality of the untargeted fecal metabolomics dataset. Unsupervised heat maps were generated using the Euclidean distance matrix with Ward clustering algorithm. For clarity and ease of interpretation only the top 50 unidentified features are displayed on the heat maps. For PND 70, a volcano plot (two group data) was used to identify significant differences between control and Test diet groups (p < 0.05; FDR p < 0.05). For PND 91, two-way ANOVA was used (p < 0.05; FDR p < 0.05). When appropriate, post hoc analysis was performed using Fisher’s PLSD with alpha set to p < 0.05. In a final step, we examined potential relationships between host physiology, the gut microbiome (phylum level), and the identified gut metabolites using stepwise multiple regression analyses. These analyses were run on the normalized microbiome/metabolomics data and examined relationships between sleep (NREM and REM) and alpha diversity data that have been previously published^7^. Differences were considered significant when p < 0.05, unless otherwise noted.

### Ethical approval

This manuscript has not been submitted to more than one journal for simultaneous consideration. All procedures were approved by the University of Colorado Animal Care and Use and all applicable guidelines for care and use of animals were followed.

## Supplementary information

Supplementary Information.

## Data Availability

All mass spectrometry data (.raw,.mzXML, and mgf files), mzMine, and Sirius files can be found in the online mass spectrometry repository Massive (http://massive.ucsd.edu) using the following accession numbers: MSV000079329 and MSV000079339.
