# Supplementary material for: Dietary prebiotics alter novel microbial dependent fecal metabolites that improve sleep
Source: Sci Rep. 2020 Mar 2;10:3848. doi: 10.1038/s41598-020-60679-y (PMC7051969; doi:10.1038/s41598-020-60679-y)
Supplement: Supplementary file 1 — Supplementary Information. [file 41598_2020_60679_MOESM1_ESM.docx]

**Dietary prebiotics alter novel microbial dependent fecal metabolites that improve sleep**

Robert S. Thompson ^1,2^, Fernando Vargas ^3,4^, Pieter C. Dorrestein ^3,4^, Maciej Chichlowski^5^, Brian M. Berg^5^, Monika Fleshner^1,2^

^1^ Department of Integrative Physiology, University of Colorado at Boulder, Boulder,
CO 80309-0354, USA; Email: [robert.s.thompson@colorado.edu](mailto:robert.s.thompson@colorado.edu) (R.S.T); [fleshner@colorado.edu](mailto:monika.fleshner@colorado.edu) (M.F.)

^2^ Center for Neuroscience, University of Colorado at Boulder, Boulder, CO 80309-0354, USA

^3^ Division of Biological Sciences, University of California, San Diego, CA 92093, USA

^4^ Collaborative Mass Spectrometry Innovation Center, Skaggs School of Pharmacy and Pharmaceutical Sciences, University of California, San Diego, CA 92093, USA

^5^ Mead Johnson Pediatric Nutrition Institute, Evansville, IN 47712, USA

***** Author to whom correspondence should be addressed; E-mail: [fleshner@colorado.edu](mailto:fleshner@colorado.edu);
Tel.: +1-303-492-1483; Fax: +1-303-492-6778. 1725 Pleasant St. 354 UCB University of Colorado at Boulder, U.S.A.

**Keywords:** neurosteroids, bile acids, ketones, hyodeoxycholic acid, galactooligosaccharides, polydextrose

Supplemental Figure 1 – Volcano plot demonstrating the features that are significantly different in control vs. Test diet groups measured in fecal samples collected on PND 70 (p < 0.05; FDR p < 0.01). See Supplemental Table 2 for details of this statistical analysis. The pink dots represent significant effects. The pink dots on the upper left denote significantly higher metabolites in the Test diet group. The pink dots on the upper right denote significantly higher metabolites in the control diet. The numbers represent *m/z* and retention times for some of these significantly different metabolites.

Supplemental Figure 2 – Two-way ANOVA demonstrating that 36 features were significantly different by diet or stress at PND 91 (p < 0.05) after adjusting for a FDR < 0.05. The specific statistical analyses of these 36 features are shown in Supplemental Table 3.


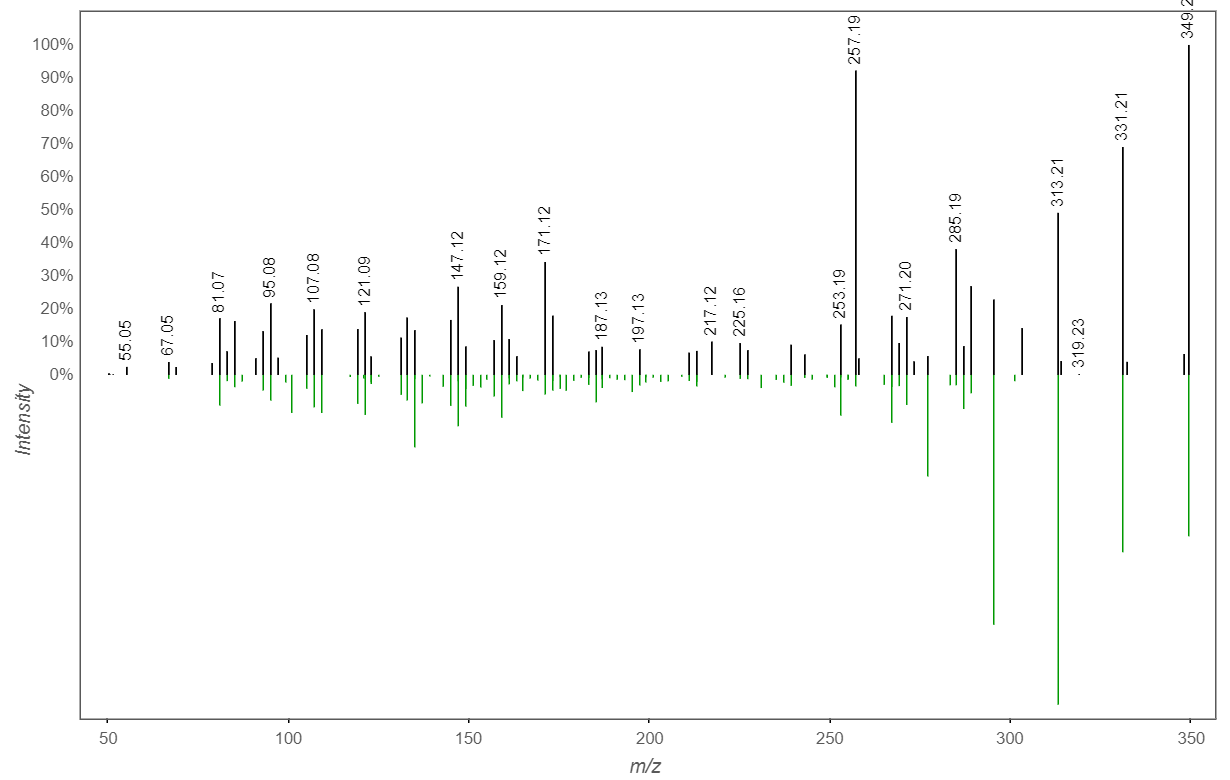


Supplemental Figure 3 – Direct MS/MS comparison between the Allopregnanolone Precursor (m/z 349.2371 rt 4.08) vs. the GNPS spectral library of 5.alpha.-Pregnane-3.alpha.,21-diol-11,20-dione. The precursor ppm error between our feature and reference standard was below 2ppm. The tandem MS2 spectra of our unidentified feature and reference library show an overlap of 46 peaks.


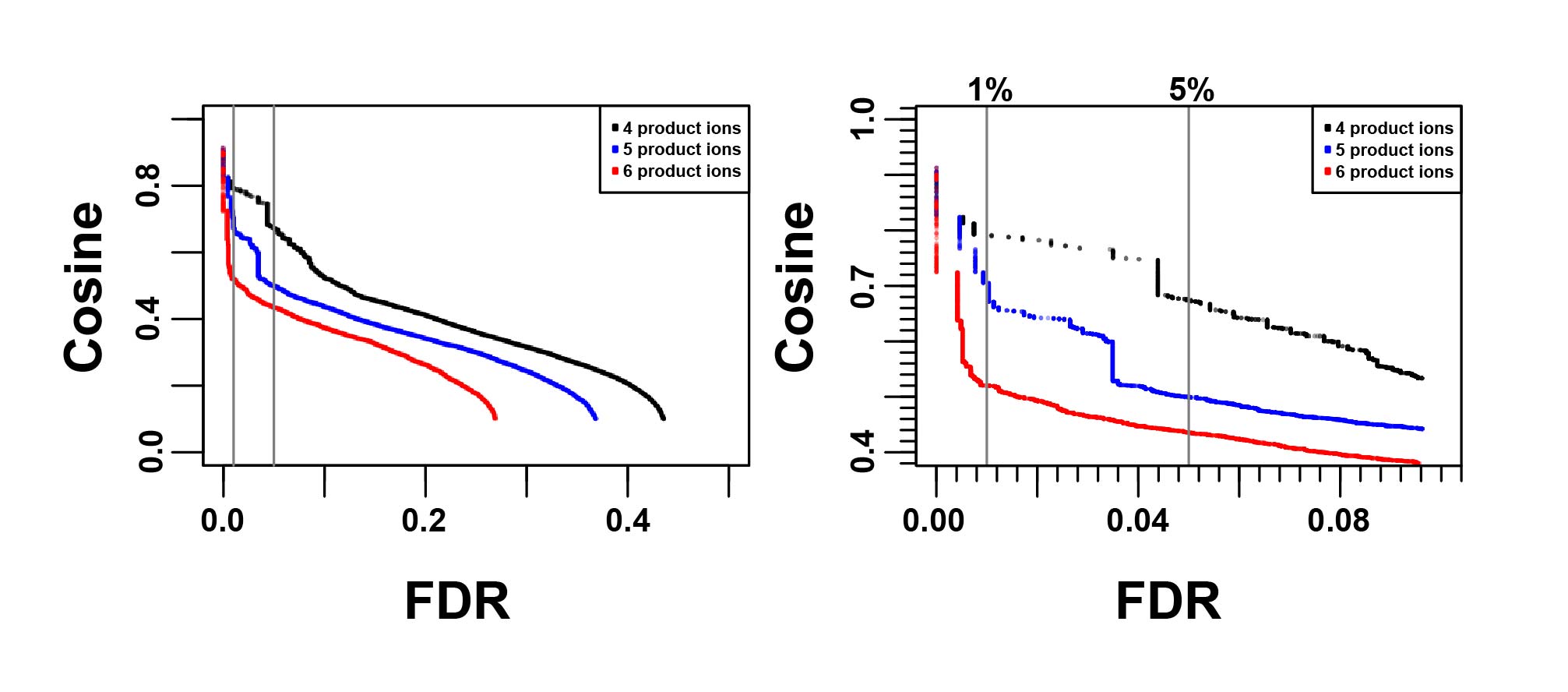


Supplemental Fig. 4 – GNPS false discovery rate plot for optimization of molecular networking parameters (FDR% vs. cosine score). To the right, zoomed in region where vertical lines indicate an FDR of 1% and 5%.

**Supplemental Table 1** – Details of LC-MS/MS parameters

| **Mzmine Parameters** |
| --- |
|  |
| **Mass Detection** |
| MS level: 1 |
| Noise level: 2E5 |
|  |
| **Chromatogram builder** |
| Min time span (min): 0.05 |
| Min height: 6E5 |
| *m/z* tolerance: 0.0005 m/z or 10 ppm |
|  |
| **Chromatogram deconvolution** |
| Local min search |
| Chromatogram threshold: 0.01% |
| Search minimum in RT (min): 0.20 |
| Minimum relative height: 0.01% |
| Minimum absolute height: 6E5 |
| Min ratio of peak top/edge: 3 |
| Peak duration range (min): 0.05-0.30 |
|  |
| **Isotope peaks grouper** |
| *m/z* tolerance: 0.005 m/z or 10 ppm |
| Retention time tolerance (min): 0.01 |
| Maximum charge: 4 |
|  |
| **Join aligner** |
| m/z tolerance: 0.0005 m/z or 10 ppm |
| Weight for m/z: 90 |
| Retention time tolerance (min): 0.3 |
| Weight for RT: 10 |
|  |
| **Peak list row filter** |
| Minimum peaks in a row: 2 |
| Minimum peaks in isotope pattern: 2 |
| Reset the peak ID: on |

**Supplemental Table 2 –** Results Table for the Volcano Plot analysis at PND 70 (Supplemental Figure 2)

| **Identified Metabolite** | **Feature (*m/z*_rt)** | | **FC** | | **log2(FC)** | **p.adjusted;**  **FDR p < 0.01** |
| --- | --- | --- | --- | --- | --- | --- |
|  | | 156.036_0.286 | | 0.03482 | -4.8439 | 6.89E-09 |
|  | | 167.5441_0.286 | | 0.022255 | -5.4897 | 1.41E-07 |
|  | | 226.1075_0.363 | | 0.06341 | -3.9791 | 0.00075567 |
|  | | 228.0572_0.288 | | 0.02801 | -5.1579 | 6.50E-15 |
|  | | 237.0625_0.291 | | 0.046724 | -4.4197 | 3.65E-06 |
| Glycerol Glucoside Derivative | | 255.1074_0.311 | | 0.0136 | -6.2003 | 1.81E-20 |
| Ethanbis(thioate) Derivative | | 293.0628_0.253 | | 0.0080589 | -6.9552 | 0.0036488 |
|  | | 309.0828_0.288 | | 0.025869 | -5.2726 | 1.51E-11 |
| Quinazolinone Analog | | 315.1111_0.355 | | 21.381 | 4.4183 | 3.17E-06 |
| Disaccharide | | 327.1282_0.331 | | 0.024425 | -5.3555 | 6.89E-16 |
| Pyrimidine Nucleotide (1) | | 339.06_0.28 | | 0.040846 | -4.6137 | 0.0037391 |
| Aminoglycoside Analog | | 342.1396_0.316 | | 0.18021 | -2.4722 | 0.0026345 |
|  | | 344.1552_0.331 | | 0.030489 | -5.0356 | 5.11E-11 |
|  | | 354.0996_0.288 | | 0.026563 | -5.2344 | 7.93E-08 |
|  | | 387.1263_0.508 | | 0.029882 | -5.0646 | 6.13E-05 |
| Disaccharide Derivative | | 417.1601_0.318 | | 0.017671 | -5.8225 | 1.81E-20 |
|  | | 435.1258_0.29 | | 0.02176 | -5.5222 | 2.64E-11 |
| Aminonucleoside Analog | | 489.1809_0.333 | | 0.040652 | -4.6205 | 3.65E-06 |
| Pyrimidine Nucleotide (2) | | 501.1125_0.276 | | 0.03786 | -4.7232 | 5.19E-06 |
| Phosphotyrosine Dipeptide | | 506.2094_0.331 | | 0.081451 | -3.6179 | 0.0010428 |
|  | | 516.1521_0.29 | | 0.042803 | -4.5462 | 8.25E-05 |

**Supplemental Table 3 –** Results Table for ANOVA at PND 91 (Supplemental Figure 3)

| **Identified Metabolite** | | **Feature (*m/z*_rt)** | **Diet** | | **Stress** | | **Diet x Stress** | | | | |  |
| --- | --- | --- | --- | --- | --- | --- | --- | --- | --- | --- | --- | --- |
|  |  | | f.value | p.value | f.value | p.value | | | f.value | | p.value | FDR p < 0.01 |
|  | | 167.5441_0.286 | 83.717 | **< 0.0001** | 2.785 | n.s. | | 2.785 | | n.s. | | 4.55E-07 |
|  | | 183.0413_0.31 | 68.832 | **< 0.0001** | 0.007 | n.s. | | 0.007 | | n.s. | | 4.53E-06 |
| Fatty Acid Derivative | | 190.1436_0.355 | 53.101 | **< 0.0001** | 5.525 | **0.027** | | 0.284 | | n.s. | | 1.48E-05 |
|  | | 192.0466_0.283 | 1834.704 | **< 0.0001** | 0 | n.s. | | 0.000 | | n.s. | | 8.12E-22 |
|  | | 211.5521_0.285 | 27.405 | **< 0.0001** | 1.253 | n.s. | | 1.147 | | n.s. | | 0.0031816 |
|  | | 220.0686_0.276 | 43.898 | **< 0.0001** | 0.357 | n.s. | | 0.357 | | n.s. | | 0.00015308 |
|  | | 228.0572_0.288 | 160.986 | **< 0.0001** | 1.135 | n.s. | | 1.135 | | n.s. | | 1.10E-09 |
| Glycerol Glucoside Derivative | | 255.1074_0.311 | 2489.595 | **< 0.0001** | 0.009 | n.s. | | 0.009 | | n.s. | | 2.24E-23 |
|  | | 264.0672_0.293 | 159.446 | **< 0.0001** | 0.18 | n.s. | | 0.180 | | n.s. | | 1.10E-09 |
|  | | 277.0889_0.26 | 68.052 | **< 0.0001** | 0.009 | n.s. | | 0.009 | | n.s. | | 4.59E-06 |
| Ethanbis(thioate) Derivative | | 293.0628_0.253 | 70.836 | **< 0.0001** | 0.004 | n.s. | | 0.004 | | n.s. | | 3.67E-06 |
|  | | 297.1801_1.995 | 401.215 | **< 0.0001** | 3.755 | 0.064 | | 0.338 | | n.s. | | 7.08E-14 |
|  | | 309.0828_0.288 | 2853.859 | **< 0.0001** | 0.014 | n.s. | | 0.014 | | n.s. | | 6.87E-24 |
| Quinazolinone Analog | | 315.1111_0.355 | 21.481 | **< 0.0001** | 2.416 | n.s. | | 0.993 | | n.s. | | 0.0068998 |
| Ketone Abietic-type Acid | | 315.1949_5.965 | 12.165 | **< 0.0001** | 0.447 | n.s. | | 3.108 | | n.s. | | 0.0084144 |
| Ketone Steroid | | 317.2469_5.103 | 21.792 | **0.003** | 18.299 | **0.000** | | 5.412 | | **0.029** | | 0.00065162 |
| Disaccharide | | 327.1282_0.331 | 4293.447 | **< 0.0001** | 0.008 | n.s. | | 0.008 | | n.s. | | 1.29E-25 |
| Pyrimidine Nucleotide (1) | | 339.06_0.28 | 148.156 | **< 0.0001** | 0.581 | n.s. | | 0.581 | | n.s. | | 2.67E-09 |
| Aminoglycoside Analog | | 342.1396_0.316 | 110.477 | **< 0.0001** | 0.233 | n.s. | | 0.233 | | n.s. | | 5.33E-08 |
|  | | 344.1552_0.331 | 1063.334 | **< 0.0001** | 0.006 | n.s. | | 0.006 | | n.s. | | 4.96E-19 |
| Allopregnanolone Precursor | | 349.2371_4.08 | 5.982 | **0.022** | 10.262 | **0.004** | | 10.119 | | **0.004** | | 0.0038076 |
|  | | 354.0996_0.288 | 28.457 | **< 0.0001** | 0.004 | n.s. | | 0.004 | | n.s. | | 0.0035903 |
|  | | 365.0846_0.261 | 29.431 | **< 0.0001** | 0.005 | n.s. | | 0.005 | | n.s. | | 0.0030548 |
|  | | 387.1263_0.508 | 169.052 | **< 0.0001** | 1.426 | n.s. | | 1.426 | | n.s. | | 7.36E-10 |
|  | | 397.1338_0.34 | 43.369 | **< 0.0001** | 0.286 | n.s. | | 0.286 | | n.s. | | 0.00016648 |
|  | | 411.0805_0.275 | 698.369 | **< 0.0001** | 0.298 | n.s. | | 0.298 | | n.s. | | 7.25E-17 |
| Disaccharide Derivative | | 417.1601_0.318 | 3640.295 | **< 0.0001** | 0.238 | n.s. | | 0.238 | | n.s. | | 5.02E-25 |
| Oligopeptide | | 434.1883_0.316 | 130.4 | **< 0.0001** | 0.167 | n.s. | | 0.167 | | n.s. | | 1.03E-08 |
|  | | 435.1258_0.29 | 1577.953 | **< 0.0001** | 0 | n.s. | | 0.000 | | n.s. | | 4.46E-21 |
|  | | 439.1422_0.261 | 68.516 | **< 0.0001** | 0.002 | n.s. | | 0.002 | | n.s. | | 4.53E-06 |
|  | | 455.116_0.256 | 44.401 | **< 0.0001** | 0.344 | n.s. | | 0.344 | | n.s. | | 0.00014599 |
|  | | 471.3077_4.925 | 3.833 | 0.062 | 15.51 | **0.001** | | 3.833 | | 0.062 | | 0.0084144 |
|  | | 481.3645_7.08 | 1.847 | n.s. | 19.067 | **0.000** | | 4.454 | | **0.045** | | 0.0053218 |
| Aminonucleoside Analog | | 489.1809_0.333 | 2569.485 | **< 0.0001** | 0.167 | n.s. | | 0.167 | | n.s. | | 1.89E-23 |
| Pyrimidine Nucleotide (2) | | 501.1125_0.276 | 77.13 | **< 0.0001** | 1.784 | n.s. | | 1.784 | | n.s. | | 1.14E-06 |
|  | | 516.1521_0.29 | 65.792 | **< 0.0001** | 0 | n.s. | | 0.000 | | n.s. | | 6.01E-06 |
